# Indicators of Mental Health Disorder, COVID-19 Prevention Compliance and Vaccination Intentions among Refugees in Kenya

**DOI:** 10.3390/medicina58081032

**Published:** 2022-08-02

**Authors:** Abayomi Samuel Oyekale

**Affiliations:** Department of Agricultural Economics and Extension, North-West University Mafikeng Campus, Mmabatho 2735, South Africa; abayomi.oyekale@nwu.ac.za or asoyekale@gmail.com

**Keywords:** COVID-19, mental health disorder, contact prevention indicator, immune boosting indicator, refugees, Kenya

## Abstract

*Background and Objectives*: COVID-19 remains a major development challenge in many developing countries. This study analysed the effect of mental health disorder and indicators of COVID-19 preventive practices on vaccination intentions among refugees in Kenya. *Materials and Methods*: The data were the fourth and fifth waves of the High Frequency Phone Surveys on the impacts of COVID-19 that were collected by the Kenyan National Bureau of Statistics (KNBS) between May 2020 and June 2021. The data were collected from Kakuma, Kalobeyei, Dadaab and Shona camps using the stratified random sampling method. The data were analysed with random effects instrumental variable Probit regression model. *Results*: The results showed that 69.32% and 93.16% of the refugees were willing to be vaccinated during the 4th and 5th waves, respectively. The fear of dying was reported by 85.89% and 74.19% during the 4th and 5th waves, respectively. COVID-19 contact prevention and immune boosting indicators were differently influenced by some demographic and anxiety index variables, while being endogenous influenced vaccine hesitancy along with urban residence, age, knowing infected persons, days of depression, days of anxiety, days of physical reactions, members losing job, searching for jobs, accepting job offers and being employed. *Conclusions*: It was concluded that efforts to promote COVID-19 vaccination should address mental health disorder and compliance with existing COVID-19 contact and immune boosting behaviour with a focus on urban residents and youths.

## 1. Introduction

Before the emergence of COVID-19 as a pandemic of significant public health concern, the African continent was one of the global epicentres of political and tribal crises, resulting in the displacement of human populations [1]. These crises have required immediate policy interventions, given their socioeconomic and health consequences. Specifically, mental health disorder remains one of the most common health problems suffered by internally displaced persons (IDPs). COVID-19 is compounding the magnitude of this problem, due to its deleterious social and economic consequences [2,3]. In Kenya, addressing the COVID-19 pandemic necessitated mandatory economic lockdowns, many of which had significant impacts on people’s mental health [4]. Although the psychological and emotional traumas that are associated with the pandemic have significant health consequences, interventions for addressing these problems are still at best deficiently implemented [4]. As of 18 July 2022, Kenya had gone through five different COVID-19 infection waves with 336,740 positive cases and 5668 deaths [5]. Among the refugees in Kenya, the first case of COVID-19 was reported in Kakuma camp on 13 March 2020. About a year later, COVID-19 positive rates increased to 7% as of 30 April 2021, with 935 deaths among refugees [6]. More importantly, recommended preventive practices are meant to slow down the speed of viral transmission, although compliance with some of these practices may also distort the functionality of individuals’ mental sanity [7,8].

Furthermore, although the Kenyan government is enforcing compliance with COVID-19 preventive practices in public places, refugees may be hindered by several socioeconomic deprivations [9,10,11]. However, the level of residential congestion and structural lapses in some refugees’ camps in terms of rowdiness [12], low access to basic sanitation practices and a lack of some essential social services may act as significant bottlenecks in complying with recommended preventive practices [13,14]. Kenya’s Ministry of Health is also emphasizing immune system boosting behaviours with the administration of internationally approved vaccines, while adequate nutrition is being advocated for COVID-19 prevention [15]. As of 18 July 2022, 32.2% of the eligible adult population in Kenya had been fully vaccinated, while 2,421,453 people had taken the first dose of the vaccines [15]. There are no disaggregated data in Kenya showing the coverage of COVID-19 vaccines among refugees. However, refugees were not discriminated from accessing vaccines, although at the onset, preferences were given to health workers and elderly refugees that are 58 years and above [6]. However, low coverage of vaccination among refugees can be inferred from the available statistics for the counties where these camps are found. Precisely, Turkana and Garissa counties, where the majority of the refugees reside, have one of the lowest vaccine coverages in Kenya, with only 17.8% and 13.4% of fully vaccinated people, respectively, as of 18 July 2022 [6].

COVID-19-induced economic hardship may also hinder refugees’ engagements with immune system-boosting behaviours through the consumption of foods that are rich in fibres and antioxidants. More specifically, the antiviral properties of some spices and herbs, such as ginger, garlic and turmeric, against SARS-CoV-2 has been reported [16,17]. Notably, garlic contains allicin, which can combat infections of the respiratory tracts, pulmonary fibrosis, sepsis and acute injuries of the lung [18,19] because of its antiviral, antifibrotic, antioxidant, anti-inflammatory and immunomodulatory properties [18]. It has also been noted that turmeric can act as a prophylaxis against SARS-CoV-2 [20], while ginger was found to reduce the symptoms of COVID-19 [21,22,23].

Some studies have analysed the determinants of compliance with COVID-19 preventive practices. These include being a woman [24,25,26,27], being 30 years or older [24,26,27,28], education status [27,28] and existence of non-communicable diseases [28]. Some studies have also analysed the determinants of vaccine hesitancy. MacDonald [29] submitted that the reasons for vaccine hesitancy can be summarized as complacency, confidence and convenience. Other studies have found that some demographic variables have influenced vaccine hesitancy. These include being young [30,31,32,33,34], older than 40 years [35], middle age [34], attainment of tertiary education [33,35,36], low education attainment [30], gender [31,32,34,35,36], black race [30], employment status [35,36,37], marital status [36,37], income level [34,36] and religion [38]. In some other studies, not getting the influenza vaccine [30], being vaccinated against the flu [35], access to social media [39], exposure to negative information on COVID-19 vaccines [36] and low perception of infection risk [32,33] influenced vaccination intention.

It should be noted that due to data paucity, studies on the intention of refugees to be vaccinated are not well documented in the literature. Similarly, there is a dearth of studies on vaccine hesitancy and mental health disorder among refugees. The linkages between behaviour changes—in relation to the prevention of COVID-19 contacts and immune boosting compliances—and vaccine hesitancy is not yet well studied. This study seeks to fill these research gaps by analysing the effect of mental health disorder and indicators of COVID-19 preventive practices on the intention to be vaccinated. It was hypothesized that indicators of mental health disorder, COVID-19 contact prevention and immune boosting compliance do not significantly influence refugees’ intentions to be vaccinated.

## 2. Materials and Methods

### 2.1. The Data

This study used data from the panel surveys on the socio-economic impacts of COVID-19 on refugees. Similar surveys were also simultaneously implemented among the Kenyan nationals. The data were collected by the Kenyan National Bureau of Statistics (KNBS), while the World Bank, the United Nations High Commissioner for Refugees (UNHCR) and the University of California, Berkeley provided some financial and technical assistance [40]. Due to movement restrictions and social distancing, the surveys were conducted through telephone interviews. The questionnaire covered a very broad scope of households’ socioeconomic activities [40].

The sampling frame comprised refugees who were registered with UNHCR. Specifically, in Kakuma and Kalobeyei camps, the sampling frame of the UNHCR’s recently conducted Socioeconomic Surveys (SES) was used, while Dadaab and Shona camps relied on the UNHCR’s registration lists of refugees [41]. The data were collected bimonthly with telephone interviews from respondents who were 18 years or above in five waves between May 2020 and June 2021. The stratified random sampling method was used [42]. At the first stage, 1000 individuals were selected from each of the strata, except Shona. The second stage involved stratification of the selected individuals by gender and age. In Shona, all the listed households were sampled due to the smallness of the sampling frame.

Text messages were sent to selected individuals as a form of alerting them that they had been selected for a survey on the socio-economic impact of COVID-19. The data were collected with a Computer-Assisted Telephone Interview (CATI). After the baseline survey, subsequent surveys were expanded with the inclusion of new respondents in order to cater for some households that may have dropped out. During the baseline survey, 1328 households were successfully sampled. However, 1699 and 1487 households were interviewed during the 2nd and 3rd waves, respectively. During the 4th and 5th waves, 1376 and 1562 households, respectively, completed the surveys [43]. Sampling weights were generated for each wave and panel, thereby enhancing the representativeness of the survey [42].

### 2.2. Analytical Procedures and Estimated Models

The effect of mental health disorder and compliance with COVID-19 preventive practices on the intention to be vaccinated was analysed with a random effects endogenous Probit regression model. This model allows for the endogeneity test to be conducted on the indicators of compliance with COVID-19 contact prevention and immune system boosting behaviours. The model also verifies the presence of heterogeneity, given the longitudinal nature of the data. The estimated model can be specified as:

(1) $$Y_{it}=\overset{n}{\underset{k=1}{\sum }}\beta_{k}X_{it}+\overset{m}{\underset{l=1}{\sum }}\pi_{k}MH_{it}+\theta C_{it}+\omega Z_{it}+v_{t}+\epsilon_{it\;}$$

In Equation (1), the dependent variable $Y_{it}$ represents the intention to be vaccinated. This variable was coded 1 for those who indicated “yes” and 0 otherwise. An individual respondent is denoted by *i*, while the panel time is denoted by *t*. Additionally, $\beta_{k}$ represents the parameters of exogenous variables ($X_{it})\;$, $\pi_{k}$ represents the parameters of mental health disorder variables ($MH_{it})$ and θ and ω are the parameters of the endogenous regressors ($C_{it}$ and $Z_{it}$). $v_{t}$ is the random effects parameter that captures existence of heterogeneity across time and $\epsilon_{it}$ is the stochastic random error. Table 1 contains the full list of these variables and their coding formats.

$C_{it}\;$ and $Z_{it\;}$ are the two indicators of compliance with COVID-19 preventive practices, which were computed using the Principal Component Analysis (PCA). The first indicator ($C_{it}$) captures COVID-19 contact prevention. This was computed from the respondents’ answers to the questions on whether in the past one week they complied with COVID-19 preventive practices of hand washing, no hand shaking, avoidance of groups of more than 10 people, hand sanitization, covering mouth when coughing, staying home, traveling less, working less, wearing masks and stocking food at home. The second indicator ($Z_{it\;}$) captures the immune systems boosting behaviour and was computed from the responses to questions on whether in the past one week the respondents drank tea with lemon, drank warm water, ate vitamin C-rich fruits, ate garlic and fruits such as avocadoes and mangoes, ate alkaline food and drank bicarbonate. A “yes” response was coded as 1 and 0 otherwise. The use of PCA to reduce the sixteen behaviour change questions into two composite indicators ensures the avoidance of multicollinearity in the estimation of Equation (1). An anxiety index was also computed with PCA from eight anxiety-related questions which are fears of losing job, infection, dying, infecting others, being unable to provide, losing access to healthcare services, education disturbances and lockdown uncertainties.

**Figure 1:**
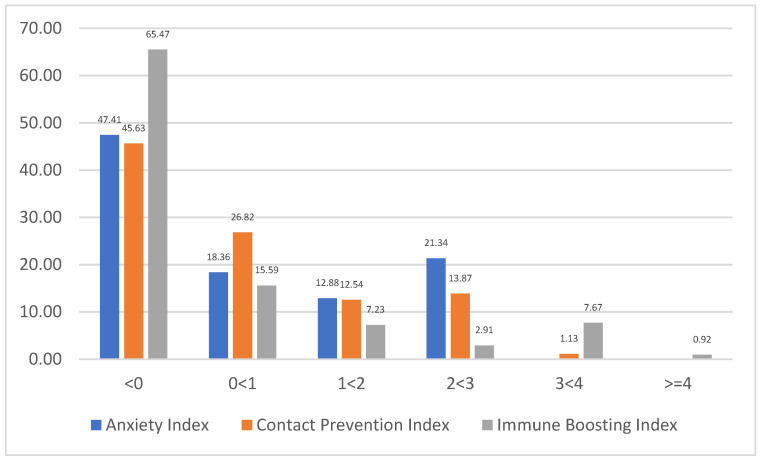
Distribution of anxiety, contact prevention and immune boosting indicators.

Table 2 shows the contributions of each component. The table shows that for the contact prevention index, the first three components account for 55.63% of the total eigenvalue. The results for the immune boosting index show that the first three components account for 70.76% of the total eigenvalue. The first three components for anxiety index account for 71.89% of the total eigenvalue. The distributions of these three indicators are presented in Figure 1. It shows that the majority of the respondents had a value of less than 1 with 65.78%, 72.46% and 81.06% for anxiety, COVID-19 contact prevention and immune boosting indicators, respectively.

The suspected endogeneity of $C_{it}$ and $Z_{it}$ in Equation (1) was addressed with some instrumental variables ($I_{it}$). These variables are expected to be correlated with $C_{it}$ and $Z_{it}\;$ but not correlated with the dependent variable ($Y_{it})$. The endogenous regressor models are specified as:

(2) $$C_{it}=\kappa +\overset{n}{\underset{k=1}{\sum }}\varphi_{k}X_{it}+\overset{m}{\underset{l=1}{\sum }}\pi_{k}MH_{it}+\overset{2}{\underset{d=1}{\sum }}\delta_{d}I_{it}+e_{it}$$

(3) $$Z_{it}=\gamma +\overset{n}{\underset{k=1}{\sum }}\eta_{k}X_{it}+\overset{m}{\underset{l=1}{\sum }}\pi_{k}MH_{it}+\overset{2}{\underset{d=1}{\sum }}\alpha_{d}I_{it}+m_{it}$$

In Equations (2) and (3), individual respondent is denoted by *i*, while t stands for time. The estimated parameters are $\;\pi_{k}$, $\varphi_{k}$, $\eta_{k}$, $\delta_{d}$, $\alpha_{d}$, κ and γ. The selected instrumental variables, $I_{it}$, are anxiety index and number of days of hopeful feelings. The basic econometric rule for instrumental variables is that they must be highly correlated with the endogenous regressors but not correlated with the dependent variable ($Y_{it}$). Additionally, $e_{it}$ and $m_{it}\;$ are the error terms. The correction for endogeneity requires that Equation (1) should be reformulated as Equation (4) with the error components of Equations (2) and (3) now included as explanatory variables. The statistical significance of the parameters of the error terms (ψ  and τ) in Equation (4) implies that endogeneity is a problem. The estimated model is specified as:

(4) $$Y_{it}=\overset{n}{\underset{k=1}{\sum }}\beta_{k}X_{it}+\overset{m}{\underset{l=1}{\sum }}\pi_{k}MH_{it}+\theta C_{it}+\omega Z_{it}+\psi e_{it}+\tau m_{it}+v_{t}+b_{it}$$

STATA 17 was used for data analyses, and it computes the value for rho, which denotes the proportion of the total variance that is accounted for by the panel level variance. The likelihood ratio test statistic for rho being equal to zero (*p* < 0.05) was also provided, and this allows us to accept or reject the null hypothesis of absence of significant heterogeneity (rho = 0). Multicollinearity among the independent variables and heteroscedasticity were tested with the variance inflation factor (VIF) and Breusch–Pagan test, respectively.

## 3. Results

### 3.1. Vaccination Intentions and Demographic Characteristics

The results in Table 3 show the distribution of the respondents’ selected demographic characteristics in the 4th and 5th data waves and their intentions to be vaccinated. It shows that during the 4th wave, only 69.32% were willing to be vaccinated. However, as expected due to increase in vaccine confidence as time passed, a significant increase occurred in the 5th wave when 93.16% of the respondents were willing to be vaccinated. It also reveals that in both data waves, the majority of the respondents were from rural areas. Additionally, male respondents constituted 51.35% and 50.06% in the 4th and 5th data waves, respectively. The results on educational attainments of the refugees show that the majority of them had no formal education with 38.13% and 33.48% in the 4th and 5th waves, respectively. However, tertiary education was reported by the lowest proportions of the respondents with 4.97% and 6.06% in the 4th and 5th waves, respectively. The distribution of respondents’ age shows that more than half of the respondents were between 20 and 39 years of age.

### 3.2. Refugees’ Exposure to Anxiety and Mental Health Disorders

**Figure 2:**
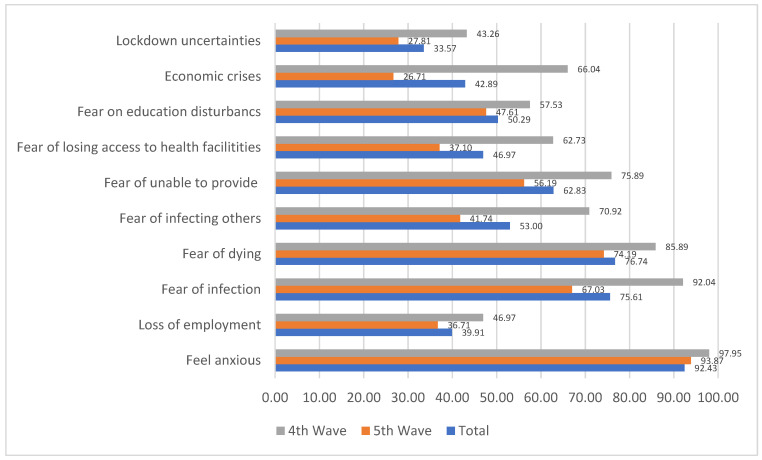
Different forms of anxiety experienced.

**Figure 3:**
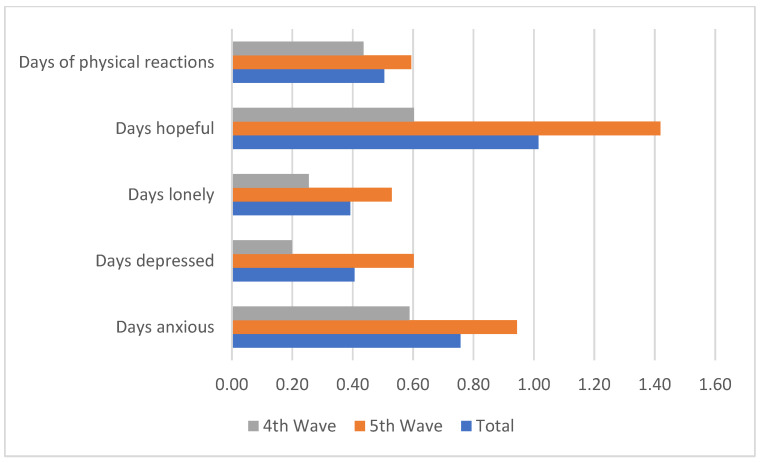
Average number of days with specific experience of mental health problems.

Figure 2 shows the distribution the different forms of emotional problems that respondents experienced. It revealed that in line with expectation, the proportions of the respondents that experienced these problems declined between the 4th and 5th waves. The results also revealed that anxiety in respect of the pandemic prevailed among most of the respondents in the 4th and 5th waves with 97.95% and 93.87%, respectively. The fears of dying from the disease and being infected were reported by 85.89% and 92.04% of the respondents in the 4th wave, while it declined, respectively, to 74.19% and 67.03% in the 5th wave. The fear of economic crises declined from 66.04% in the 4th wave to 26.71% in the 5th wave. Figure 3 also reveals the average number of days that the respondents experienced some selected mental health problems. It shows that between the 4th and 5th wave, the average number of days when they experienced loneliness, depression and anxiety increased. Similarly, there was an increase in the average number of average hopeful days between the 4th and 5th waves.

### 3.3. Determinants of COVID-19 Contact Prevention and Immune Boosting Indicators

Table 4 shows the determinants of refugees’ COVID-19 contact prevention and immune boosting indicators. The results were generated with Ordinary Least Square (OLS) regression. Evidenced by low variance inflation factor (VIF), multicollinearity was not a problem among the included variables. The model also produced a good fit of the data, given the statistical significance of the F-statistics. In line with expectation, the estimated parameter for urban residence in the immune boosting model shows statistical significance (*p* < 0.01) with positive sign. This indicates that taking other variables constant, urban residents among the refugees had higher immune boosting indices when compared with those from rural areas. None of the education dummy parameters showed statistical significance in the immune boosting model, while primary education parameter shows statistical significance (*p* < 0.01) with negative sign in the contact prevention model. This result indicates that taking other variables constant, the refugees with primary education had lower contact prevention indices, when compared with those with no formal education.

Contrary to expectation, the parameter of age in the immune boosting model is with negative sign and shows statistical significance (*p* < 0.01). This implies that as age increases, the immune boosting indicator decreases, holding other variables constant. Contrary to expectation, the parameter of knowing infected person is with negative sign and statistically significant (*p* < 0.01). This implies that refugees that knew COVID-19-infected persons had lower contact prevention indices. The parameters of the number of market visitations in the two models are statistically significant (*p* < 0.01). The results imply that increase in the number of market visitations increased contact prevention indicator but decreased immune boosting indicator. Additionally, the parameter of number of people that were interacted with on the day of interview by the respondents shows statistical significance (*p* < 0.01) in the immune boosting model. This implies that an increase in the number of people interacted with will reduce the immune boosting index.

In the immune boosting model, the parameters of employment-related variables—members losing jobs, members searching for jobs, members accepting job offers and members being employed—are all with negative signs and statistically significant (*p* < 0.01). The results indicate that the respondents whose households had members that lost their jobs, searched for jobs, accepted job offers and are employed have lower immune boosting indicator. The results in the COVID-19 contact prevention model show that the parameters of variables on members losing jobs, members searching for jobs, members accepting job offers and members being employed are statistically significant (*p* < 0.01). These results indicate that households where members lost jobs, accepted job offers and are employed had higher COVID-19 contact prevention index, while those where members searched for jobs had lower values.

Furthermore, in line with expectation, the parameters of anxiety index in the two models are with positive signs and statistically significant (*p* < 0.01). These results show that as the anxiety indicator increases, COVID-19 contact prevention and immune boosting indicators increased. The results also reveal that the parameters of number of days nervous and depressed are statistically significant (*p* < 0.01) in the two models, although those in the contact model are negatively signed while the ones for immune boosting model are positively signed. In the COVID-19 contact prevention model, the parameters of the number of lonely days, days of physical reactions and days hopeful are statistically significant (*p* < 0.01). However, days of physical reactions shows statistical significance in the immune boosting behaviour (*p* < 0.05).

### 3.4. Determinants of COVID-19 Vaccination Intention

The results in Table 4 show the estimated parameters with random effects Probit model. It shows that the model produced a good fit for the data given that the Wald Chi-Square statistics shows statistical significance (*p* < 0.01). However, the likelihood ratio test statistics is statistically insignificant (*p* > 0.05). This implies a significant absence of random effects and that conventional Probit model will produce the same results. It should also be noted that the parameters of the residuals from the COVID-19 contact prevention and immune boosting models are both statistically significant (*p* < 0.01). Based on expectation, these results are confirming endogeneity of the COVID-19 contact prevention and immune boosting indicators. The implication is that estimated parameters would be inconsistent and biased if endogeneity had not been corrected.

The results show that the COVID-19 contact prevention and immune boosting indicators are statistically significant (*p* < 0.01). Therefore, the study’s hypotheses in line with these variables should be rejected. Specifically, in line with expectation, an increase in the immune boosting indicator increases the probability of willingness to take the vaccines. On the other hand, expectedly, increase in the COVID-19 contact prevention will reduce the probability of willingness to take the vaccines.

The parameter of urban residence is statistically significant with negative sign (*p* < 0.01). This implies that taking other variables as constant, urban refugees had significantly lower probability of willing to be vaccinated. However, unexpectedly, none of the education dummy parameters shows statistical significance (*p* > 0.10). In line with expectation, increase in refugees’ age will significantly increase the probability of willing to be vaccinated (*p* < 0.05). The parameter of knowing a COVID-19-infected person is statistically significant (*p* < 0.01). Therefore, contrary to expectation, the refugees that knew someone that had been previously infected with COVID-19 had a significantly lower probability of willing to be vaccinated. An increase in the number of times a refugee visited markets in the past seven days significantly increased the probability of willingness to take the vaccines (*p* < 0.01). This is in line with expectation.

The results also showed that all the employment-related variables in the model show statistical significance (*p* < 0.01). Expectedly, refugees from households where members had lost their jobs during COVID-19 pandemic had significantly higher probability of willingness to take the vaccines. However, the respondents from households where members were searching for jobs had significantly lower probability of willingness to take the vaccines. In addition, expectedly, the respondents from households where members accepted job offers and are employed during the pandemic had significantly higher probabilities of willingness to take the vaccines. The results further show that the study’s hypothesis in relation to mental health cannot be accepted because three of the four mental health disorder variables show statistical significance (*p* < 0.01). Precisely, in line with expectation, an increase in the number of days that the respondents were nervous, depressed and with physical reactions increased the probability of willingness to take the vaccines.

## 4. Discussion

The proportions of the refugees that were willing to be vaccinated increased substantially between the fourth and fifth waves. This is expected and reflects the rapid increase in the confidence that people developed in the vaccines over time. Although COVID-19 vaccines confronted a significant wave of misinformation and disinformation that initially slowed acceptability [44,45], variations still exist in their acceptability across countries and regions of the world [46,47,48]. In Kenya, hesitancy towards COVID-19 vaccines was 63.3% among some teachers in January 2021 [49]. However, in a Kenyan national survey that was conducted in June 2021, vaccine hesitancy declined to 19.38% [50]. The growing understanding and perception of COVID-19 vaccine safety has facilitated its reliability ratings, thereby enhancing the willingness of adults and children to get vaccinated [51]. However, this does not completely nullify the reality of fear as many people relapse into pandemic fatigue that reduced their confidence in further engagement in some COVID-19 preventive methods such as social distancing and sanitation [52]

The results further reveal the different forms of mental health problems that were reported by refugees. Although, in line with expectation, the proportions of the households that reported these problems declined between the fourth wave (15 January–25 March) and the fifth wave (29 March–13 June), the intensity seemed to have worsened going by some increases in the average number of days for which mental distresses were reported. One of the fundamental impacts of COVID-19 pandemic is mental health disorder, which manifests in the form of depression, anxiety, distress and low self-esteem. The World Health Organization (WHO) reported that the prevalence of anxiety and depression increased by about 25 percent within the first year of the pandemic. The major forms of the stress include loneliness resulting from isolation after infection, fear of being infected or death, financial hardships and job losses [53].

Although urban residence significantly increases the immune boosting indicator, its association with willingness to be vaccinated is negative. Residents in urban areas may possess some advantages over their rural counterparts in access to sufficient incomes to purchase immune boosting fruits and spices. In addition, by the virtue of their expected high education level, urban households are expected to possess significant awareness on the immune boosting properties of those plant products. More diverse findings have been reported on the association between urban residence and willingness to take COVID-19 vaccines. The finding is contrary to some previous studies [54,55] that reported a higher probability of vaccine acceptance among urban residents. However, the results are in tandem with some studies that found urban residents to have lower probability of willing to take the vaccine [54,56].

The results revealed that as age of the refugees increases, immune boosting indicator decreases. However, an increase in the age of refugees increased the probability of willing to get vaccinated. In some previous studies, age had been found to have different impacts on COVID-19, the knowledge of the disease and vaccine hesitancy. It had been found that older people had more knowledge of COVID-19 transmission methods [54]. Some studies have reported that older people have higher probability of willing to take COVID-19 vaccines [24,26,27,28,30,31,32,33,34]. In some other studies, older people had lower probability of willing to take COVID-19 vaccines [35,56,57]. The understanding of the relationship between age and COVID-19 vaccine hesitancy is often presented based on the vulnerability of old people to the disease and an individual’s evaluation of the tendency to develop some chronic adverse reactions from the vaccines.

Knowing a COVID-19-infected person(s) is expected to act as double confirmation on the existence of the disease and severity of its symptoms. Ideally, the impact this can have on engagement with preventive practices may be positive or negative, depending on the ultimate evaluation of an individual’s perceptions of infection risks. In this study, knowing an infected person decreased the COVID-19 contact prevention index and probability of willingness to be vaccinated. Conceptually, these findings are contrary to expectations [58], but should be seen in light of the fact that individuals’ assessment of associated risks of the pandemic can vary from one circumstance to another.

Another important variable that was explored is the number of visits to the markets by the refugees in the past seven days. This variable captures the intensity of compliance with avoidance of crowds as part of the measures to control the spread of COVID-19 [59,60]. In this study, increase in the number of times a refugee visited the market increased COVID-19 contact prevention index and decreased the immune boosting index. The number of people interacted with also reduced immune boosting indicator. However, this variable increased the probability of willing to take the vaccines. The findings reveal a higher compliance with COVID-19 contact prevention practices by those who would unavoidably visit public places such as the markets. Accordingly, the number of market visitation is also positively associated with willingness to be vaccinated. This is in tandem with the conceptual view that individuals who consider themselves to be at a higher risk of contracting the virus would show more positive attitude towards vaccination and other preventive/protective behaviour [61,62].

One of the vital impacts of the pandemic was on labour market engagement because many businesses folded during the pandemic. It had been estimated that about 740,000 Kenyans lost their jobs during the pandemic [63]. With the service sector leading job creation, the huge impacts of COVID-19 on job losses in Kenya is not surprising [64]. All the labour-market-related variables that were included, except job searching showed positive impacts on COVID-19 contact prevention indicator and willingness to get vaccinated. These variables however had negative influence on immune boosting indicator. The findings are buttressing the role of labour market engagements in COVID-19 contact preventive practices and willingness to be vaccinated. The results are clearly underpinning acceptability and high level of COVID-19 safety compliance among refugees that lost their jobs, newly got employed or had employed members. These results may also reflect the tendency of employed people to avoid being sick from COVID-19 [35,36,37] and mandatory vaccination policy being suggested for some workers [65].

Increase in the days of physical reactions (such as coughing and flu) increased contact prevention indicator but decreased immune boosting indicator. These findings showed that refugees were able to engage in more COVID-19 contact prevention practices with less utilization of practices that could boost their immune systems. It also reveals compliance with some essential practices that are necessary for preventing transmission of COVID-19 [66,67]. In addition, some of the indicators of mental health disorder are significantly influencing contact prevention and immune boosting indicators. Specifically, contact prevention indicators increased with the number of days that the refugees felt depressed and anxious although these variables have negative influence on the immune boosting indicator. Furthermore, as expected, probability of willingness to take the vaccines also increased with days of physical reactions, depression and anxiety. These finding are implying that although some mental problems are associated with COVID-19, they also promote willingness to be vaccinated against the pandemic.

This study acknowledges some data and estimation issues that could have influenced the results. First, the study utilized secondary data and some mental health variables that could be found within the surveys. The absence of properly scaled variables that can diagnose a wider range of mental health disorders constitutes a major limitation. In addition, the variables that were utilized to compute the immune boosting indicator did not properly probe into the intensity of utilization and consumption frequency which are the hallmarks for realizing the full benefits from some of those fruits and culinary herbs and spices. Finally, although this study used data for two waves, the major feature of panel data in terms of dropped households and addition of new ones presents a situation of an unbalanced panel. This can introduce some sensitivity in comparing statistical estimators across the different survey waves.

## 5. Conclusions

This study assessed the effect of indicators of mental health disorder and COVID-19 prevention indicators on Kenyan refugees’ willingness to be vaccinated. Although several studies have focused on the interplay of socioeconomic and demographic characteristics on vaccine hesitancy, very little is currently known on the role of mental-health-related problems and COVID-19 preventive indicators in respect of contact prevention and immune system boosting. This present study also presents analyses for a refugee population that had been rarely study in any COVID-19-related empirical research.

Based on the findings of the study, the following recommendations are made. Although willingness to be vaccinated increased over time among the refugees, efforts to guarantee wider acceptability of the vaccines should be put in place with an emphasis on dissolution of raging waves of vaccine misinformation. Such efforts should focus on the promotion of COVID-19 vaccines’ safety in a transparently sincere manner. There is also the need to properly integrate documentation and treatment of mental-health-related problems, as part of the comprehensive initiative to fight COVID-19. This is fundamentally essential because an inability to properly integrate these problems within the forefront of COVID-19 management underestimates the consequences of the pandemics. Although the Kenyan government has made efforts in ensuring that contact with COVID-19 is minimized with some strict measures that were among others targeted at social distancing, sanitation and disinfection of hands and environment, conscientious efforts should focus on the role of immune system strengthening in addressing a pandemic of global relevance such as COVID-19. In addition, efforts to address vaccine hesitancy among refugees should be directed at urban residents and young people.

## Figures and Tables

**Table 1:** Descriptive statistics of the selected explanatory variables.

Explanatory Variables	Frequency	%	Mean	Std. Dev.	Min	Max
Vaccination intention (Yes = 1, 0 otherwise)	2388	81.81	0.8181	-	0	1
Urban residence (Yes = 1, 0 otherwise)	619	21.21	0.2121	-	0	1
No education (Yes = 1, 0 otherwise)	1034	35.43	0.3543	-	0	1
Primary Education (Yes = 1, 0 otherwise)	914	31.31	0.3131	-	0	1
Secondary Education (Yes = 1, 0 otherwise)	809	27.7	0.2770	-	0	1
Tertiary Education (Yes = 1, 0 otherwise)	163	5.57	0.0557	-	0	1
Age of respondent (years)	-	-	35.1881	12.4804	18	85
Male respondent (Yes = 1, 0 otherwise)	1479	50.67	0.5067	-	0	1
Know infected person (Yes = 1, 0 otherwise)	248	8.5	0.0850	-	0	1
Number of market visits	-	-	2.3340	2.3403	0	23
Number of people interacted with today	-	-	4.8123	5.8020	0	50
Member lost jobs (Yes = 1, 0 otherwise)	67	2.3	0.0230	-	0	1
Members searched for jobs (Yes = 1, 0 otherwise)	1198	41.04	0.4104	-	0	1
Members accepted job offer (Yes = 1, 0 otherwise)	1282	43.92	0.4392	-	0	1
Members are employed (Yes = 1, 0 otherwise)	787	26.96	0.2696	-	0	1
Immune boosting indicator	-	-	0.0000	1.4455	−0.8732	5.7879
Contact prevention index	-	-	0.0000	1.5860	−2.6992	3.2721
Days nervous	0.7568	75.68	0.7568	1.2883	0	5
Days depressed	0.4063	40.63	0.4063	0.9432	0	5
Days lonely	0.3919	39.19	0.3919	0.9972	0	5
Days of physical reactions.	0.505	50.5	0.5050	1.0122	0	5
*Endogenous variables*						
Contact prevention model residuals	-	-	0.0000	1.1888	−3.8144	3.9913
Immune boosting model residuals	-	-	0.0000	0.9798	−2.8548	4.9486
*Instrumental variables*						
Days hopeful	1.0154	101.54	1.0154	1.6276	0	5
Anxiety index	-	-	0.0000	1.9478	−3.2889	2.8727

**Table 2:** Eigenvalues and contributions of each component to estimated parameter.

	Contact Prevention Index		Immune Boosting Index		Anxiety Index	
Component	Eigenvalue	Proportion	Eigenvalue	Proportion	Eigenvalue	Proportion
Comp1	2.5155	0.2515	2.08949	0.3482	3.79379	0.4215
Comp2	1.90593	0.1906	1.16148	0.1936	1.74384	0.1938
Comp3	1.14161	0.1142	0.994806	0.1658	0.932152	0.1036
Comp4	0.960899	0.0961	0.825939	0.1377	0.739208	0.0821
Comp5	0.80813	0.0808	0.50399	0.0840	0.497656	0.0553
Comp6	0.656557	0.0657	0.424288	0.0707	0.411025	0.0457
Comp7	0.577343	0.0577			0.353058	0.0392
Comp8	0.547964	0.0548			0.28519	0.0317
Comp9	0.453091	0.0453			0.24408	0.0271
Comp10	0.432982	0.0433				

**Table 3:** Selected demographic characteristics and vaccination intentions.

	Wave 4 (*n* = 1369)		Wave 5 (*n* = 1550)		All (*n* = 2919)	
Vaccination Intention	Frequency	%	Frequency	%	Frequency	%
Agree to vaccination	949	69.32	1444	93.16	2393	81.98
Disagree to vaccination	425	31.04	106	6.84	531	18.19
Rural	1077	78.67	1223	78.90	2300	78.79
Urban	292	21.33	327	21.10	619	21.21
Gender						
Male	703	51.35	776	50.06	1479	50.67
Female	666	48.65	774	49.94	1440	49.33
Education						
None	522	38.13	519	33.48	1041	35.66
Primary	405	29.58	506	32.65	911	31.21
Secondary	374	27.32	432	27.87	806	27.61
Tertiary	68	4.97	94	6.06	162	5.55
Age						
<20	54	3.94	63	4.06	117	4.01
20 < 30	493	36.01	529	34.13	1022	35.01
30 < 40	389	28.41	449	28.97	838	28.71
40 < 50	246	17.97	287	18.52	533	18.26
50 < 60	125	9.13	143	9.23	268	9.18
>=60	62	4.53	79	5.10	141	4.83

**Table 4:** Determinants of Vaccination Intention Based on Random Effects Probit Model.

Variables	Coefficient	Std. Error	z Stat.	*p* > z
*Demographic characteristics*				
Urban resident	−0.4607	0.1141	−4.04	0.000
Age of respondent	0.0098	0.0039	2.53	0.012
Male respondent	0.1159	0.0740	1.57	0.117
Primary Education level	−0.1191	0.1141	−1.04	0.297
Secondary Education level	0.0508	0.1049	0.48	0.628
Tertiary Education level	−0.0865	0.1829	−0.47	0.636
*Social interactions*				
Know infected person	−0.4596	0.1567	−2.93	0.003
Times visited markets in past 7 days	0.4474	0.0806	5.55	0.000
People interacted with today	−0.0003	0.0071	−0.04	0.965
*Employment*				
Household member lost jobs	1.5144	0.4438	3.41	0.001
Members searching for jobs	−0.2383	0.1044	−2.28	0.022
Members accepted job offer	1.4829	0.3823	3.88	0.000
Members are employed	1.5289	0.2411	6.34	0.000
*COVID-19 preventive indicators*				
Immune systems boosting indicator	0.9087	0.3359	2.71	0.007
COVID-19 contact prevention indicator	−0.9113	0.2652	−3.44	0.001
*Anxiety and mental health*				
Number of days nervous, anxious	0.3624	0.0835	4.34	0.000
Number of days depressed	0.2437	0.0836	2.92	0.004
Number of days lonely	−0.0785	0.0500	−1.57	0.116
Number of days of physical reactions	0.2009	0.0557	3.60	0.000
*Residuals*				
Residuals from Contact Index Regression Model	1.1381	0.2674	4.26	0.000
Residuals from Immune Index Regression Model	−1.4775	0.3406	−4.34	0.000
Constant term	−1.4490	0.5339	−2.71	0.007
lnsig2u	−3.2124	2.7805		
sigma_u	0.2006	0.2789		
Rho	0.0387	0.1034		
Number of observations	2910			
Wald chi2(21)	218.81			
Prob > chi2	0.0000			
LR test of rho = 0: chibar2(01) = 0.14				

## Data Availability

The data for this study were collected by the United Nations High Commission for Refugees (UNHCR). The author obtained permission to download the data from https://microdata.unhcr.org/index.php/catalog/296/ (accessed on 20 June 2022).
